# An Epstein-Barr Virus Encoded Inhibitor of Colony Stimulating Factor-1 Signaling Is an Important Determinant for Acute and Persistent EBV Infection

**DOI:** 10.1371/journal.ppat.1003095

**Published:** 2012-12-27

**Authors:** Makoto Ohashi, Mark H. Fogg, Nina Orlova, Carol Quink, Fred Wang

**Affiliations:** Department of Medicine, Brigham & Women's Hospital and Department of Microbiology and Immunobiology, Harvard Medical School, Boston, Massachusetts, United States of America; Northwestern University, United States of America

## Abstract

Acute Epstein-Barr virus (EBV) infection is the most common cause of Infectious Mononucleosis. Nearly all adult humans harbor life-long, persistent EBV infection which can lead to development of cancers including Hodgkin Lymphoma, Burkitt Lymphoma, nasopharyngeal carcinoma, gastric carcinoma, and lymphomas in immunosuppressed patients. BARF1 is an EBV replication-associated, secreted protein that blocks Colony Stimulating Factor 1 (CSF-1) signaling, an innate immunity pathway not targeted by any other virus species. To evaluate effects of BARF1 in acute and persistent infection, we mutated the BARF1 homologue in the EBV-related herpesvirus, or lymphocryptovirus (LCV), naturally infecting rhesus macaques to create a recombinant rhLCV incapable of blocking CSF-1 (ΔrhBARF1). Rhesus macaques orally challenged with ΔrhBARF1 had decreased viral load indicating that CSF-1 is important for acute virus infection. Surprisingly, ΔrhBARF1 was also associated with dramatically lower virus setpoints during persistent infection. Normal acute viral load and normal viral setpoints during persistent rhLCV infection could be restored by Simian/Human Immunodeficiency Virus-induced immunosuppression prior to oral inoculation with ΔrhBARF1 or infection of immunocompetent animals with a recombinant rhLCV where the rhBARF1 was repaired. These results indicate that BARF1 blockade of CSF-1 signaling is an important immune evasion strategy for efficient acute EBV infection and a significant determinant for virus setpoint during persistent EBV infection.

## Author Summary

Epstein-Barr virus (EBV) is a herpesvirus that persistently infects nearly all humans by adulthood. Acute and persistent phases of EBV infection are associated with a variety of human diseases, including infectious mononucleosis and cancer. To investigate how EBV interacts with the host to successfully establish acute and persistent infection, we combined the power of the rhesus macaque animal model for EBV infection with genetic engineering of the EBV-related herpesvirus, or lymphocryptovirus (LCV), that naturally infects rhesus macaques. We created a recombinant rhLCV carrying a mutated EBV BARF1 homologue, a replication-associated viral protein that is secreted and blocks Colony Stimulating Factor-1 (CSF-1) signaling, a cytokine important for innate immunity. Oral inoculation of rhesus macaques showed that the virus' ability to block CSF-1 was important for achieving the normally high viral loads during acute infection, and surprisingly, was also needed to establish normal levels of virus infection, or viral setpoint, during persistent infection. These studies show that virus-mediated interruption of innate immunity is critical for both acute and persistent phases of EBV infection. Understanding how EBV successfully infects humans and how the natural history of EBV infection can be disrupted will aid in development of vaccines to prevent EBV-associated diseases.

## Introduction

Acute Epstein-Barr virus (EBV) infection is the most common cause of Infectious Mononucleosis (IM). Once infected, EBV persists in rare peripheral blood lymphocytes for the life of the host [1]. Almost all humans are persistently EBV infected by adulthood, and persistent EBV infection is almost always asymptomatic as long as host immunity is intact. The number of virus-infected peripheral blood lymphocytes, or virus setpoint, during persistent EBV infection is stable over time [2]. However, in rare instances, persistent infection leads to EBV-associated cancers such as Hodgkin lymphoma, Burkitt lymphoma, nasopharyngeal carcinoma, gastric carcinoma, and B cell lymphomas in immunocompromised people [1]. How virus setpoints are established, how cancer develops from persistent EBV infection, and how virus setpoints affect cancer development remain important unanswered questions.

EBV infection of peripheral blood lymphocytes in tissue culture has provided detailed knowledge for the molecular events associated with B cell growth transformation and virus replication [1]. Less well understood are the dynamics of virus infection in humans where EBV must penetrate the oral mucosa and amplify itself during acute infection to gain access and establish persistent, latent infection in the peripheral blood B cell compartment. The lack of small animal models that can accurately reproduce the biology of acute and persistent EBV infection has limited investigation of the relationship between acute and persistent phases of infection, as well as identification of determinants for EBV infection outcomes.

Most non-human primates are infected with a herpesvirus that is closely related to EBV and shares the biologic features of EBV infection [3]. Infection of rhesus macaques with their EBV-related herpesvirus, or lymphocryptovirus (LCV), provides a unique opportunity for experimental studies of EBV pathogenesis [4]. The rhLCV genome is colinearly homologous to EBV [5], and the biology of natural rhLCV infection is similar, if not identical, to EBV infection of humans, eg oral transmission, acute viral load with establishment of life-long persistent infection, and development of virus-induced malignancies after immunosuppression [3]. Additionally, the rhesus macaque cellular and humoral immune responses to rhLCV infection closely mirror those of EBV-infected humans [6]–[9]. rhLCV-naive macaques can be experimentally infected by oral inoculation, reproducing the natural route of transmission followed by acute, persistent, as well as malignant LCV infection in association with Simian Immunodeficiency Virus (SIV) infection that appears indistinguishable from EBV infection of healthy and Human Immunodeficiency Virus (HIV)-infected humans [4], [10].

Experimental animal models are important for dissecting the consequences of host-pathogen interactions, especially viral proteins that modify innate or adaptive immune responses. EBV encodes at least five proteins capable of modifying host immunity to EBV infection. All five viral proteins are expressed during replicative, but not latent, infection indicative of their importance for disruption of host immune responses to viral replication. Three EBV proteins interfere with Major Histocompatibility Complex (MHC) class I mediated antigen presentation (BNLF2a, BILF1, and BGLF5; [11]–[13]), whereas a fourth EBV protein, BCRF1, is an IL-10 homologue [14].

A fifth protein, BARF1, is secreted from infected cells and acts as a soluble Colony Stimulating Factor-1 (CSF-1) receptor to block CSF-1 mediated signaling, a pathway of innate immunity not known to be targeted by other viruses [15]. CSF-1 is a cytokine important for macrophage production, differentiation, and function, as well as for bone and placental development [16]. BARF1 is a 29 kda monomer which forms a novel hexameric ring to bind CSF-1 [17] and interferes with CSF-1 signaling by multiple pathways including sequestration of cytokine, interference with the receptor-binding surface, and induction of a conformational change preventing interaction with its native receptor [18]. BARF1 functionally blocks CSF-1-induced macrophage proliferation [15] and interferon alpha production from mononuclear cells in vitro [19]. Tissue culture studies with EBV and rhLCV mutants have established that BARF1 is not essential for viral replication or B cell immortalization [19], [20]. The evolution of BARF1 homologues in Old World non-human primate LCV [5], but non-essential role for BARF1 in vitro indicates that BARF1 has an important role in natural host infection. To better understand the role of BARF1 in EBV infection, we mutated the BARF1 homologue in rhLCV to study the effect on rhesus macaque infection.

## Results

### Oral Inoculation of rhLCV-Naïve Rhesus Macaques with a Recombinant rhLCV Encoding a Defective rhBARF1: *Acute Infection*

In order to test the importance of rhBARF1-mediated CSF-1 blockade in acute and persistent rhLCV infection, three rhLCV-naïve rhesus macaques were orally inoculated with 10^6^ transforming units (TU) of a recombinant rhLCV (ΔrhBARF1) carrying a truncated rhBARF1 previously shown to be incapable of blocking CSF-1-mediated signaling [20]. Successful penetration of the oral mucosa and invasion of the peripheral blood was evaluated by reverse-transcriptase-mediated PCR (RT-PCR) amplification for the rhLCV homologue of the small EBV-encoded RNAs (rhEBER) in peripheral blood mononuclear cells (PBMC). EBER are abundantly expressed in LCV-infected B cells, with approximately 100,000 copies per cell [21], making rhEBER RT-PCR an extremely sensitive assay for the detection of rare rhLCV-infected cells in the peripheral blood [22].

PBMC were isolated from weekly peripheral blood samples and distributed into multiple aliquots, with the number of cells per aliquot dependent on total PBMC yield per blood draw (eg, 3, 5, or 10×10^6^ PBMC/aliquot). RNA was extracted from individual aliquots, and rhEBER and GAPDH RT-PCRs were performed. Aliquots were scored as positive or negative after gel electrophoresis and Southern blot hybridization of PCR products with radiolabelled gene-specific oligonucleotide probes.

**Figure 1:**
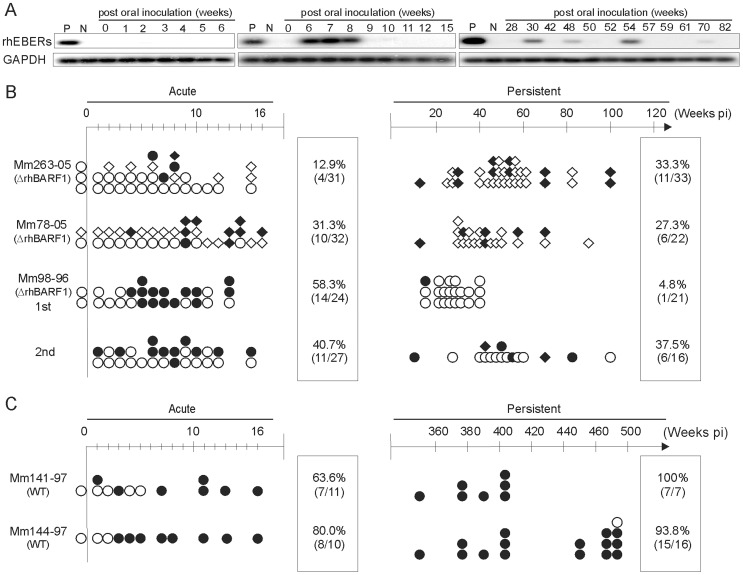
rhEBER expression in PBMC after experimental oral inoculation of rhesus macaques with rhLCV. **A**) Representative southern blot hybridization following RT-PCR amplification for rhEBER (top panel) and GAPDH (lower panel) from PBMC RNA in Mm263-05 after ΔrhBARF1 rhLCV inoculation. RNA from a rhLCV-infected B cell line and LCV-negative BJAB cells served as positive (P) and negative (N) controls respectively. **B**) Schematic representation of positive (filled symbols) and negative (open symbols) rhEBER RT-PCR results on PBMC aliquots from three rhLCV-naïve rhesus macaques inoculated with ΔrhBARF1 rhLCV (Mm263-05, Mm78-05, and Mm98-96). Aliquots containing 3 or 5×10^6^ PBMC are shown as circles, and aliquots containing 10×10^6^ PBMC are shown as diamonds. The percentage of positive samples during the acute (weeks 1–16 post-inoculation (pi)) and persistent (weeks >16 pi) phases of infection are shown in boxes to the right. Mm98-96 was orally re-challenged with ΔrhBARF1 rhLCV (2^nd^) 40 weeks after the first (1^st^) oral inoculation. **C**) Schematic representation of rhEBER RT-PCR from PBMC aliquots after oral inoculation with WT rhLCV in 2 rhLCV-naïve rhesus macaques (Mm141-97 and Mm144-97).

Representative RT-PCR assays after ΔrhBARF1 rhLCV oral inoculation of a naïve rhesus macaque (Mm263-05) are shown in Figure 1A. Single PBMC aliquots from weeks 0–6 tested negative for rhEBER (Figure 1A, left panel), but repeat testing of a second week 6 aliquot tested positive along with aliquots from week 7 and 8 (Figure 1A, middle panel). rhEBER RT-PCR testing of PBMC aliquots from later time points were occasionally positive, eg week 30, 48, 54, and 70 as shown in Figure 1A (right panel).

The results of rhEBER RT-PCR assays for all Mm263-05 PBMC aliquots tested are summarized in Figure 1B (open symbols = negative results, closed symbols = positive results). In the acute phase (defined as weeks 0–16), 4 of 31 aliquots (12.9%) were positive, and viral RNA was detectable between 6 to 8 weeks post-oral inoculation, a time frame similar to the acute viral load (weeks 3–10) reported previously by DNA PCR after experimental inoculation with wild type (WT) rhLCV [10]. Oral inoculation of two other rhLCV-naïve rhesus macaques with ΔrhBARF1 rhLCV (Mm78-05 and Mm98-96 (1^st^)) also resulted in intermittently positive PBMC aliquots by rhEBER RT-PCR testing during the acute phase (31.3% and 58.3% positive respectively).

These results were compared to inoculation with WT rhLCV by using archived PBMC aliquots from rhesus macaques orally inoculated with 10^6^ TU of WT rhLCV (Mm141-97 and Mm144-97 [10]). As shown in Figure 1C, rhEBER RT-PCR assays became positive from 1–3 weeks after oral WT rhLCV inoculation, and then stayed almost uniformly positive for months to years after oral inoculation. Thus, an intact rhBARF1 was not essential for successful penetration of the oral mucosa and entry into peripheral blood, but the ΔrhBARF1 rhLCV-infected animals had a lower level of viral load during the acute phase compared to infection with WT rhLCV.

### Oral Inoculation of rhLCV-naïve Rhesus Macaques with a Recombinant rhLCV Encoding a Defective rhBARF1: *Persistent Infection*

To determine whether ΔrhBARF1 rhLCV could establish persistent infection, PBMC samples from multiple time points >16 weeks post-oral inoculation were tested by rhEBER RT-PCR. In all three ΔrhBARF1 rhLCV-inoculated animals, rhEBER was detectable in intermittent PBMC aliquots (Figure 1B). 11 of 33 PBMC aliquots tested positive (33.3%) in the persistent phase for Mm263-05, 6 of 22 PBMC aliquots tested positive (27.3%) for Mm78-05, and 1 of 21 positive PBMC aliquots tested positive (4.8%) for Mm98-06. Mm98-96 was also orally rechallenged with ΔrhBARF1 rhLCV at 40 weeks post-inoculation. There was an increase in PBMC aliquots testing positive by rhEBER RT-PCR during the acute (40.7%) and persistent (37.5%) periods following re-inoculation. Overall, the rate of positive rhEBER RT-PCR testing during the persistent phase of ΔrhBARF1 rhLCV infection (4.8%–37.5%) was much lower than the nearly universal rhEBER RT-PCR positive results during persistent infection with WT rhLCV (93.8%–100%). Of note, the PBMC aliquots tested from Mm141-97 and Mm144-97 contained 3–5×10^6^ cells (Figure 1C, circles), whereas many of the PBMC aliquots from ΔrhBARF1 rhLCV-infected animals contained 10×10^6^ PBMC (Figure 1B, diamonds). Thus, the rhBARF1-defective rhLCV was capable of establishing persistent infection, but the intermittent detection, even when using aliquots with larger numbers of cells, indicated a lower frequency of virus-infected cells in the peripheral blood of ΔrhBARF1 rhLCV-infected animals during persistent infection.

### rhLCV Encoding a Defective rhBARF1 Establishes a Lower Virus Setpoint during Persistent Infection

**Figure 2:**
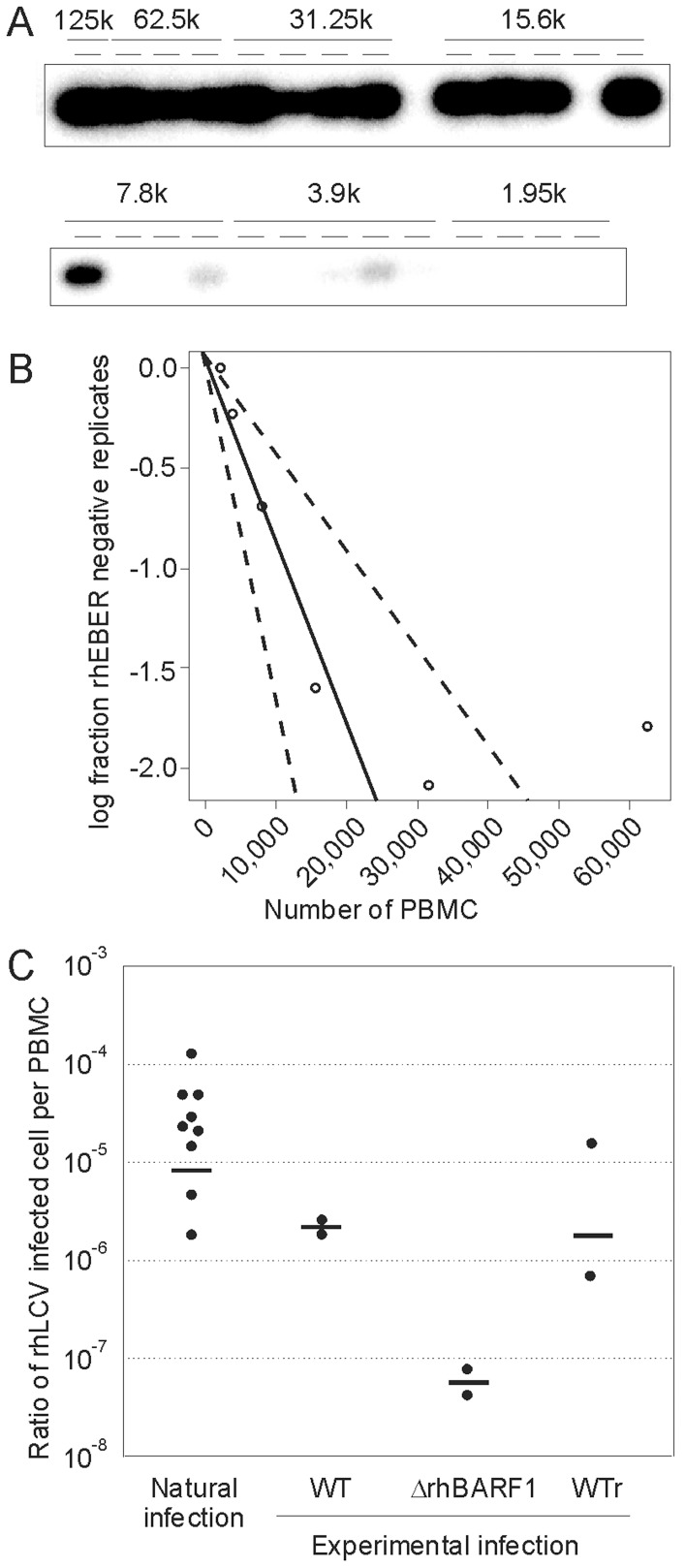
Limiting dilution analysis using rhEBER RT-PCR to determine frequency of rhLCV-infected cells during persistent infection. **A**) rhEBER RT-PCR results of serially diluted PBMC aliquots (cell number per aliquot indicated above blot) from a single blood draw during persistent infection in a rhesus macaque naturally infected with rhLCV. **B**) A log-fraction plot of limiting dilution analysis for rhEBER RT-PCR data in panel A derived by extreme limiting dilution analysis [23]. The slope of the solid line is the log-active cell fraction. The 95% confidence interval is shown by the dotted lines. **C**) Summary of rhLCV virus setpoints during persistent infection in naturally infected animals or animals experimentally infected with WT rhLCV, recombinant rhLCV containing a mutated rhBARF1 (ΔrhBARF1), or recombinant rhLCV where the rhBARF1 ORF was repaired (WTr). Geometric means are represented by solid horizontal lines.

To more precisely quantitate the frequency of virus-infected cells in the peripheral blood, or virus setpoint, during persistent rhLCV infection, limiting dilution analysis with rhEBER RT-PCR was performed on PBMC collected from a single time point. PBMC were aliquoted to provide multiple replicates with decreasing numbers of cells, eg 5 replicates with 10^6^ cells, 5 replicates with 0.5×10^6^ cells, etc. A representative experiment using PBMC obtained from a rhesus macaque in the conventional colony, ie naturally infected with rhLCV, is shown in Figure 2A. All replicates with 125000, 62500, and 31250 PBMC/replicate tested positive, whereas 4/5, 2/4, 1/5, and 0/4 tested positive from replicates with 15600, 7800, 3900, and 1950 PBMC/replicate respectively. By Poisson distribution and application of extreme limiting dilution analysis (ELDA) to account for the small number of replicates [23], a frequency of 1 infected cell per 11,240 PBMC (with a 95% confidence interval between 5,976 and 21,143) was estimated (Figure 2B). Results from 9 random, naturally infected rhesus macaques showed an average frequency of 1 infected cell per 115,943 PBMC with a range of 1 in 8,342–570,565 during persistent infection (Figure 2C; natural infection).

To determine whether experimental infection with WT rhLCV could establish persistent infection at levels similar to natural infection, limiting dilution assays were performed using PBMC from Mm141-97 and Mm144-97 obtained years after experimental oral inoculation with WT rhLCV. These assays showed a virus setpoint of 1 infected cell per 558,765 and 377,815 PBMC respectively (Figure 2C; WT), only slightly lower than the virus setpoints established in natural infection. Thus, experimental rhLCV infection can reproduce virus setpoints comparable to that seen in naturally infected animals. Differences between experimental and natural infections, eg different viral strains, viral titer of the oral challenge, and potential for multiple challenge/reinfections, may contribute to the slight differences in virus setpoints.

PBMC from ΔrhBARF1 rhLCV-infected animals were tested by limiting dilution and rhEBER RT-PCR to determine virus setpoints during persistent infection. Few replicates tested positive for rhEBER expression (1/25 for Mm263-05, 2/33 for Mm98-96, and 0/12 for Mm78-05) even though the highest numbers of PBMC/replicate were increased to 2×10^6^, 1.5×10^6^, and 2×10^6^ PBMC/replicate respectively. Poisson distribution using ELDA indicated that the frequencies of rhLCV-infected cells in Mm263-05 and Mm98-96 were on the order of 1 in 24.5 million and 1 in 12.8 million PBMC respectively (Figure 2C, ΔrhBARF1). No reliable estimate can be made for Mm78-05 in the absence of a positive replicate, but the number of replicates and PBMC/replicate used indicated the actual frequency of rhLCV-infected cells was less than 1 in 3.8 million PBMC. The precision of these analyses could theoretically be improved by using larger numbers of PBMC/replicate, but this could not be done given the limit of blood allowed per single phlebotomy. The analysis indicated that the virus setpoint during persistent infection was approximately 100 fold lower with ΔrhBARF1 rhLCV (mean of 1 in 15.3 million PBMC for Mm263-05 and Mm98-96) compared to animals with natural rhLCV infection (mean of 1 in 115,943 PBMC).

**Figure 3:**
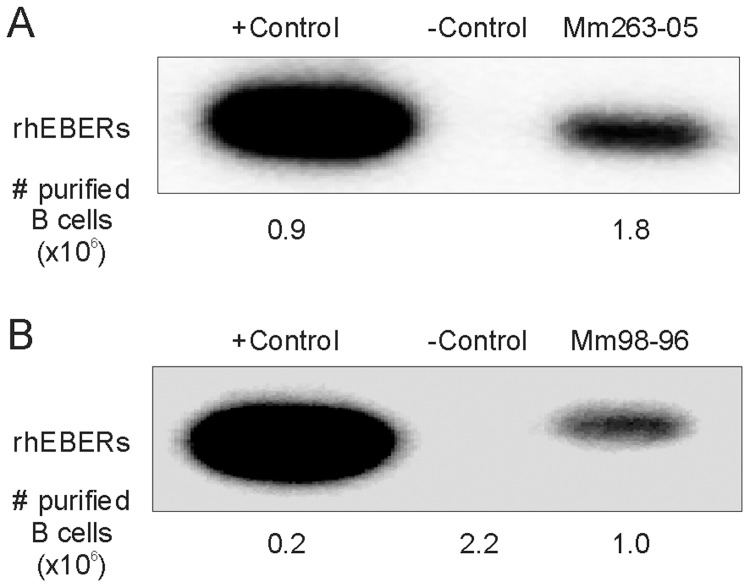
Detection of rhLCV infection in purified B cells during persistent infection after oral inoculation with ΔrhBARF1 rhLCV. rhEBER RT-PCR using RNA from affinity purified CD20+ B cells from ΔrhBARF1 rhLCV-infected animals Mm263-05 (**A**) and Mm98-96 (**B**). The number of affinity purified B cells used is shown below each panel. Positive and negative control RNAs were derived from purified B cells from a WT rhLCV-infected (Mm141-97) and LCV-negative BJAB in panel A, and purified B cells from a naturally infected rhesus macaque and rhLCV-naïve rhesus macaque in panel B.

To support the hypothesis that oral inoculation with ΔrhBARF1 rhLCV resulted in persistent infection at a very low frequency, we purified CD20+ B cells from PBMC to test whether rhEBER could be more easily detected if we increased the number of target cells in a single sample. Affinity purification of B cells should increase the sensitivity of detection by enriching for rhLCV-infected cells and by eliminating dilution of rhEBER RNA by nucleic acids from non-B cells. In Figure 3A, rhEBER RT-PCR testing was performed with RNA isolated from BJAB cells (a LCV-negative B lymphoma cell line), 0.9×10^6^ B cells from Mm141-97, and 1.8×10^6^ B cells from Mm263-05. rhEBER were strongly detected in RNA from Mm141-97, an animal experimentally infected with WT rhLCV, and not in RNA from the negative control BJAB. A weaker, but positive, rhEBER signal was obtained using the RNA from Mm263-05, experimentally infected with ΔrhBARF1 rhLCV. A similar experiment is shown in Figure 3B where rhEBER were readily detected in RNA from 0.2×10^6^ B cells of a naturally rhLCV-infected animal, not detected in RNA from 2.2×10^6^ B cells of a rhLCV-naïve animal, and detected in RNA from 1×10^6^ B cells of Mm98-96, experimentally infected with ΔrhBARF1 rhLCV. The ability to detect rhLCV infection using larger numbers of B cells confirmed the presence of persistent infection in ΔrhBARF1 rhLCV-infected animals. The results were also consistent with both the limiting dilution studies and intermittent rhEBER positivity in PBMC aliquots over time, indicating a low virus setpoint after oral inoculation with ΔrhBARF1 rhLCV.

### Scarce Adaptive Immune Responses in Animals Infected with rhLCV Encoding a Defective rhBARF1

We asked whether the low virus setpoints in ΔrhBARF1 rhLCV-infected animals were associated with strong immune responses that could drive virus setpoints lower than usual. Surprisingly, ΔrhBARF1 rhLCV infection was associated with delayed or undetectable adaptive humoral and cellular immune responses. Serum antibody responses against the small viral capsid antigen (rhsVCA; rhBFRF3) were undetectable from Mm98-96 and 78-05 for the entire study period, even though rhsVCA serum antibody responses are ubiquitous in macaques with natural rhLCV infection, as they are in EBV-infected humans [22]. In addition, no serum antibody responses from Mm98-96 and 78-05 were detected against recombinant rhLCV lytic infection proteins rhBRLF1, rhBZLF1, rhBMRF1, rhBILF2, rhBALF4, and rhBALF2 even though we previously described that serum antibody responses against these antigens can be detected in 61%, 83%, 90%, 95%, 100%, and 100% of naturally infected animals respectively [8]. rhLCV-specific T cell responses could not be detected from Mm78-05 at weeks 23 and 125 post-inoculation nor from Mm98-96 at weeks 63 and 177 post-inoculation by in vitro expansion using autologous rhLCV-immortalized B cell lines, as previously described [6]. Thus, any rhLCV-specific adaptive immune response to ΔrhBARF1 rhLCV infection in these animals was below the level of detection by these assays and much lower than the level of adaptive responses typically present in naturally infected animals.

**Figure 4:**
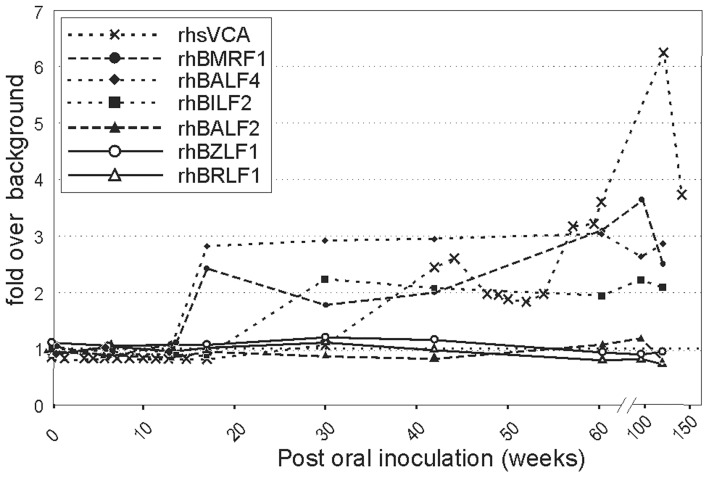
Delayed serologic responses to rhLCV antigens after oral inoculation with ΔrhBARF1 rhLCV. The presence of serum antibodies in Mm263-05 was determined by enzyme immunoassay against an immunodominant peptide for the rhLCV rhBFRF3 small viral capsid antigen (rhsVCA) and purified recombinant proteins for rhBZLF1, rhBRLF1, rhBMRF1, rhBALF2, rhBALF4, and rhBILF2. Results are expressed as fold increase in optical density over background wells with no serum.

rhLCV-specific serologic responses were detected after ΔrhBARF1 rhLCV inoculation in Mm263-05, but they were delayed compared to infection with WT rhLCV. As shown in Figure 4, serum antibodies to rhsVCA in Mm263-05 were detected beginning at week 42, whereas rhsVCA-specific antibodies are typically detected by week 7 after experimental inoculation with WT rhLCV [10]. rhsVCA-specific antibodies in Mm263-05 rose over time and remained positive during the study through week 142, consistent with persistent rhLCV infection. Serologic responses were also detected against rhBMRF1 and rhBALF4 at week 18, both responses appearing delayed compared to weeks 6 and 8 after infection with WT rhLCV [8]. Mm263-05 also developed serologic responses to rhBILF2 at week 30, but rhBILF2 serologic responses to WT rhLCV are variable and less predictable [8]. rhLCV-specific T cells were not detected in Mm263-05 at week 137 post-inoculation after stimulation with autologous rhLCV-immortalized B cells. Thus, even though some serologic responses were detected in Mm263-05, they were still abnormal relative to that expected for WT rhLCV infection, they developed after the low ΔrhBARF1 rhLCV setpoint had been established, and T cell responses were not detected. These results indicated that the low virus setpoints established during ΔrhBARF1 rhLCV persistent infection were more likely due to a defect in establishing persistent infection, as opposed to ongoing suppression of virus setpoints by a strong immune response.

### rhLCV with a Defective rhBARF1 Can Establish a Virus Setpoint Similar to WT rhLCV in an Immunosuppressed Host

**Figure 5:**
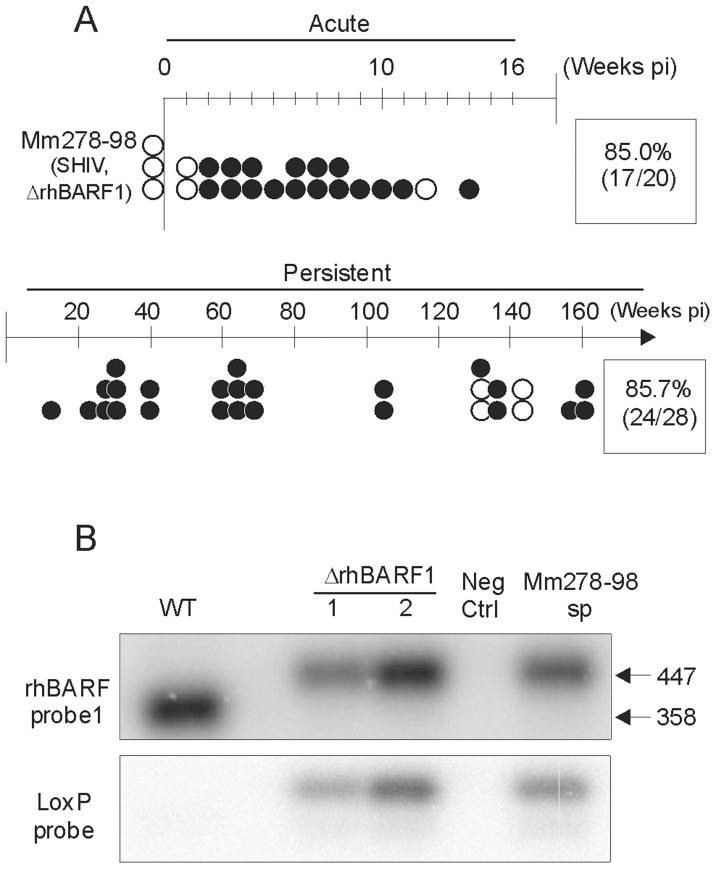
Experimental oral inoculation of an immunosuppressed, rhLCV-naïve rhesus macaque with ΔrhBARF1 rhLCV. A ) rhEBER RT-PCR results from PBMC of a SHIV-immunosuppressed rhesus macaque (Mm278-98) after oral inoculation with ΔrhBARF1 rhLCV. **B**) Molecular signature for ΔrhBARF1 rhLCV DNA in a spontaneously growing B cell line recovered from Mm278-98 PBMC 3 weeks after oral inoculation with ΔrhBARF1 rhLCV (Mm278-98sp). rhBARF1 DNA was PCR amplified from control B cell lines containing WT rhLCV, ΔrhBARF1 rhLCV, or no LCV (neg ctrl) and hybridized with either a rhBARF1 or loxP probe to differentiate the WT rhLCV genome signature (358 bp, rhBARF1+, loxP−) from the ΔrhBARF1 rhLCV signature (447 bp, rhBARF1+, loxP+). The rhLCV genome recovered in a spontaneous B cell line from the blood after oral inoculation of Mm278-98 has a ΔrhBARF1 rhLCV signature (Mm278-98sp).

If rhBARF1 were required for immune evasion in vivo, we predicted that the blunted acute viral load and low virus setpoint in persistent infection after ΔrhBARF1 rhLCV inoculation could be normalized by immunosuppressing the host. A rhLCV-naïve macaque (Mm278-98) was first immunosuppressed by Simian/Human Immunodeficiency Virus (SHIV) infection followed by oral inoculation with 10^6^ TU of ΔrhBARF1 rhLCV. As shown in Figure 5, rhEBER expression was detected in PBMC beginning at week 2 post-inoculation and in the vast majority of PBMC aliquots tested from the acute phase (17/20; 85%). Similarly, rhEBER expression was detected in the vast majority of PBMC aliquots tested from the persistent phase of infection (24/28; 85.7%). The high frequency of PBMC aliquots testing positive for rhEBER expression during acute and persistent phases after ΔrhBARF1 rhLCV inoculation of an immunosuppressed host was more similar to experimental infection with WT rhLCV (Mm141-97 and Mm144-97) than to ΔrhBARF1 rhLCV infection of immunocompetent hosts (Mm263-05, Mm98-96, and Mm78-05).

Limiting dilution analysis of Mm278-98 PBMC taken at week 159 post-inoculation showed that approximately 1 in 195,725 PBMC were infected, a level more comparable to animals experimentally infected with WT rhLCV (1 in 115,943; Figure 2C) than immunocompetent animals infected with ΔrhBARF1 rhLCV (1 in 15.3 million; Figure 2C). These results indicated that SHIV-induced immunosuppression can rescue the phenotype of ΔrhBARF1 rhLCV infection and that ΔrhBARF1 rhLCV was capable of establishing normal rhLCV setpoints under certain conditions.

rhLCV-specific adaptive immune responses were detected in Mm278-98. Serum antibodies to the rhsVCA were detected beginning at week 41 post-oral inoculation, and serum antibodies to, rhBALF2 (≥ week 6), rhBMRF1 (≥ week25), and rhBALF4 (≥ week 25), but not rhBZLF1, rhBRLF1, and rhBILF2, were detected. CD8+ T cell responses specific for rhEBNA-2, rhEBNA-3C, rhBRLF1, rhBLLF2, and rhBZLF1 were also detected in T cell lines derived from PBMC drawn at weeks 77, 81, 88, 97, and 102 weeks post-oral inoculation. Thus, ΔrhBARF1 rhLCV could readily induce adaptive immune responses in immunosuppressed hosts where it achieved higher viral loads during acute infection and higher virus setpoints during persistent infection.

### Recovery of rhLCV Encoding a Defective rhBARF1 from the Peripheral Blood after Experimental Oral Inoculation

To determine whether virus in the infected host retained the same molecular genotype of the inoculating virus, we cultured PBMC in vitro for spontaneous outgrowth of virus-immortalized B cells. No spontaneously growing B cell lines could be derived from immunocompetent animals infected with ΔrhBARF1 rhLCV (Mm263-05, Mm98-96, and Mm78-05), likely due to the very low frequency of infected cells. A spontaneously growing B cell line was derived from PBMC collected at week 3 post-inoculation in the immunosuppressed host (Mm278-98).

The rhLCV strain isolated from the peripheral blood of Mm278-98 was identified by PCR amplification of rhBARF1 viral DNA from the spontaneous B cell line. ΔrhBARF1 rhLCV DNA has a unique loxP scar sequence in the rhBARF1 coding sequence where the Bacterial Artificial Chromosome (BAC) vector sequences were initially inserted and later excised [20]. Cre-mediated excision of the BAC vector sequences from the recombinant virus leaves 89 nucleotides from the loxP scar sequence in rhBARF1 resulting in a frame shift and premature termination of the rhBARF1 coding sequence. Thus, the 89 additional nucleotides and loxP scar sequence at the rhBARF1 locus provide a unique molecular signature for ΔrhBARF1 rhLCV.

PCR amplification across the BAC vector insertion site distinguishes ΔrhBARF1 rhLCV from WT rhLCV, as shown in Figure 5B. A 358 bp PCR fragment could be amplified from a cell line infected with WT rhLCV, and it hybridized with an internal, radiolabelled rhBARF1 probe (Figure 5B, top panel, WT). PCR amplification from ΔrhBARF1 rhLCV-immortalized cell lines derived in tissue culture resulted in a slightly larger, 447 bp DNA fragment that not only hybridized with the rhBARF1 probe (Figure 5B, upper panel, ΔrhBARF1 1 & 2), but also a loxP probe (Figure 5B, lower panel). Similarly, PCR amplification from the spontaneous Mm278-98 B cell line (Mm278-98sp) resulted only in the larger 447 bp PCR product and contained the loxP scar sequence. These studies provide formal proof that the same molecular clone used to orally inoculate Mm278-98 was capable of penetrating the oral mucosa and infecting cells in the peripheral blood where it could be recovered as a spontaneous B cell line in vitro.

### Repair of the rhLCV rhBARF1 Open Reading Frame Restores Virus Setpoints in Immunocompetent Hosts

**Figure 6:**
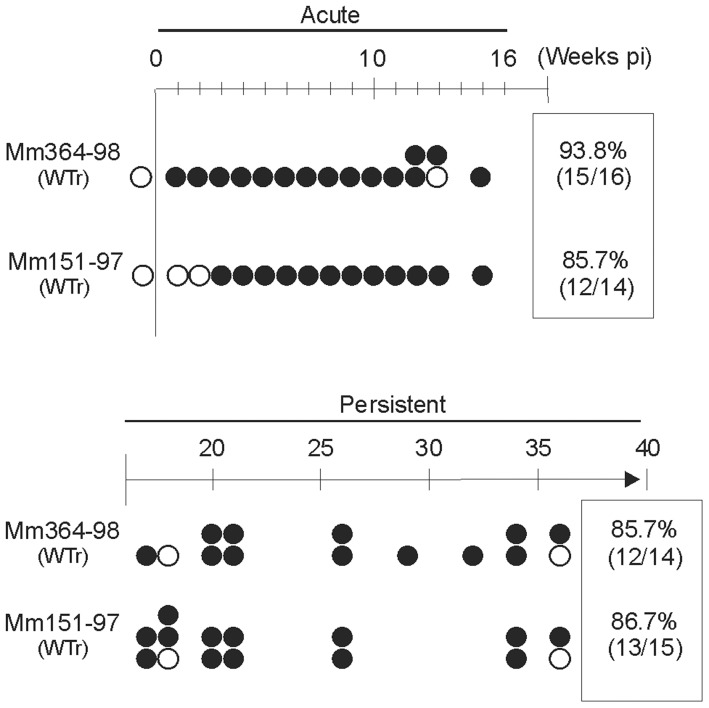
Experimental infection with a recombinant rhLCV clone where the rhBARF1 ORF in ΔrhBARF1 rhLCV was repaired. Schematic representation of rhEBER RT-PCR results from PBMC aliquots during acute and persistent phases of infection after oral inoculation of 2 rhLCV-naïve rhesus macaques (Mm364-98 and Mm151-97) with the recombinant rhLCV containing a repaired rhBARF1 ORF (WTr).

To demonstrate that the rhBARF1 mutation was responsible for the ΔrhBARF1 rhLCV phenotype, the rhBARF1 ORF in the ΔrhBARF1 rhLCV BAC was restored [20], and the wild type repaired virus (WTr) was used to orally inoculate immunocompetent rhLCV-naïve rhesus macaques, Mm364-98 and Mm151-97. As shown in Figure 6, rhEBER were detected at weeks 1-3 post-inoculation with positive results in 15/16 (93.8%) and 12/14 (85.7%) of aliquots containing 3–5×10^6^ PBMC during acute infection. 12/14 (85.7%) and 13/15 (86.7%) PBMC aliquots were positive during persistent infection. The kinetics of rhEBER expression in PBMC after oral inoculation with WTr was comparable to experimental infection with WT rhLCV (Figure 1C; Mm141-97 and Mm144-97) from which the BAC was originally derived.

Limiting dilution analysis showed virus setpoints of 1 in 43,317 and 1 in 1,404,968 PBMC for Mm364-98 and Mm151-97 at weeks 19 and 29 respectively, levels comparable to both natural rhLCV infection and experimental WT rhLCV infection (Figure 2C). Repeat testing at weeks 24 and 32 post-inoculation showed frequencies of 1 in 62,589 and 1,105,494 PBMC for Mm364-98 and Mm151-97 respectively indicating that the virus setpoint was relatively stable during persistent infection in experimentally infected rhesus macaques and that persistent infection was achieved by 19 weeks post-oral inoculation. Reversal of the abnormal phenotype by repair of the rhBARF1 ORF indicated that the loss of CSF-1 blocking ability was linked to the lower viral load during acute infection and lower virus setpoints during persistent infection with ΔrhBARF1 rhLCV.

## Discussion

These experiments break new ground in two major areas by taking studies of EBV gene function into the context of a natural host and by exploring viral blockade of CSF-1 signaling, an immune evasion strategy not previously described for any other virus. Our studies indicate that viral amplification during acute EBV infection is susceptible to CSF-1-induced immune responses since mutating rhBARF1 resulted in blunting of acute viral load during the first 16 weeks after oral inoculation. In addition, we found a dramatic lowering of the virus setpoint during persistent infection when the virus was incapable of blocking CSF-1-induced immune responses. This anti-viral effect could be reversed by either immunosuppressing the host via SHIV infection or by restoring CSF-1 blocking ability to the rhBARF1 ORF.

An important role for BARF1 during acute EBV infection might be predicted since it is expressed during lytic replication [24] and lytic replication is assumed to be an important mechanism for viral amplification during acute infection. However, there is little published evidence to support this assumption. Anti-viral drugs, such as acyclovir, that can block EBV replication have shown no significant efficacy in clinical trials with IM patients [25], [26]. One interpretation could be that lytic replication is not important in acute EBV infection. Another interpretation may be that anti-viral therapy is initiated too late in IM patients to be effective, since viral amplification through lytic replication may have already occurred by the time patients present with symptoms and are recruited into a clinical trial. Human epidemiologic studies indicate that viral inoculation occurs approximately 6 weeks before IM symptoms develop [27], and our animal studies show that virus usually becomes detectable in the peripheral blood within 3 weeks after oral inoculation. Similarly, the failure to find robust viral replication in tonsillar epithelial cells of IM patients may be due to the timing of the studies [28], as opposed to evidence against an important role for lytic viral replication in acute infection. Our results provide the first experimental evidence linking lytic EBV replication to viral amplification during acute EBV infection.

The mechanisms by which CSF-1 enhances immune control of acute EBV infection remain to be identified. CSF-1 induces differentiation and maturation of monocytes into active phagocytes in tissue culture [16], and CSF-1 administration in vivo can increase blood monocytes and tissue resident macrophages [29]. Thus, EBV-infected cells during acute infection may be particularly sensitive to CSF-1-activated phagocytic cells and tissue macrophages. CSF-1 also acts on other components of the immune system that may contribute to its anti-viral effect. CSF-1 increases conventional and plasmacytoid dendritic cells that produce interferon alpha and activate NK cells [30], [31], and CSF-1 has been reported to mobilize and enhance NK cytolytic activity [32]. Thus, it is tempting to speculate that BARF1 may have been acquired as a unique EBV strategy to evade NK cells. There are multiple lines of evidence linking EBV susceptibility to NK cells. Patients with X-Linked Lymphoproliferative Syndrome (XLP) suffer from fatal IM or lymphomas upon primary EBV infection, but do not have unusual susceptibility to other microbial pathogens [33]. In XLP, mutations in the signaling lymphocytic activation molecule (SLAM)-associated protein (SAP) prevent activation of NK cell cytotoxicity against EBV-infected B cells [34]. Severe combined immunodeficiency (SCID) mice engrafted with human PBMC depleted of NK cells are more susceptible to fatal lymphoproliferation of infused EBV-infected B cells than non-depleted controls, indicating a role for NK cells in preventing outgrowth of infected B cells [35]. B cell immortalization by EBV infection in tissue culture is also sensitive to NK cells [36], and in particular to tonsil-derived NK cells that may be most proximal to early viral events during acute EBV infection in humans [37]. Interestingly, HIV and SHIV infection have been associated with decreases in circulating NK cells and NK cell function [38], [39], and this may explain why SHIV-induced immunosuppression was associated with rescue of a normal viral phenotype after ΔrhBARF1 rhLCV inoculation.

The markedly decreased virus setpoint during persistent infection with ΔrhBARF1 rhLCV was surprising. Viral set points during persistent EBV infection are believed to be independent of lytic viral replication since they remain largely unchanged in patients on chronic acyclovir therapy [2], although recent data has challenged this paradigm [40]. A potential model to explain our findings with ΔrhBARF1 rhLCV may be that the virus setpoint during persistent infection is determined in part by the level of viral load during acute infection, ie the effect on virus setpoint in persistent infection is a consequence of, or dependent upon, the rhBARF1 effect in acute infection. This is similar to Herpes Simplex Virus where multiple factors during acute infection, including viral replication, multiplicity of infection, and location of replication, can contribute to the level of persistent, latent infection established in neurons [41]. Alternatively, rhBARF1 may have an additional and independent effect on persistent infection. Although BARF1 is typically expressed during lytic replication [24], BARF1 mRNA can be detected in nasopharyngeal carcinoma cells [42], [43] and EBV-immortalized B cells [44] in the absence of lytic replication opening up the theoretic possibility that BARF1 may be promiscuously expressed during persistent infection in latently infected peripheral blood B cells. Further studies will be required to understand the mechanistic basis for the low virus setpoints established by ΔrhBARF1 rhLCV, but this is the first experimental demonstration that the natural history of persistent LCV infection can be attenuated.

The failure to generate robust adaptive immune responses in hosts persistently infected with ΔrhBARF1 rhLCV was also surprising since control of latently EBV-infected B cells in humans is tightly linked to adaptive, and in particular, T cell immune responses [1]. This indicated that adaptive immune responses were not the principle driver for the low virus setpoint during persistent ΔrhBARF1 rhLCV infection. It may be more likely that ΔrhBARF1 rhLCV is a conditionally attenuated virus, ie in a healthy, immunocompetent host, it is incapable of establishing a normal virus setpoint in the face of intact CSF-1-induced immune responses, and the lower virus setpoint provides insufficient antigen expression to stimulate robust adaptive immune responses. When hosts are immunosuppressed or CSF-1 is blocked, the virus can achieve higher setpoints associated with more antigenic stimulation and easily detectable adaptive immune responses. This unusual state of persistent infection with very low virus setpoints and difficult to detect adaptive immune responses may need to be considered when evaluating vaccine studies, ie to differentiate sterilizing immunity from an altered natural history of persistent EBV infection with a very low virus setpoint.

Altering the natural history of persistent EBV infection is likely to be an important component for an effective EBV vaccine for IM and for EBV-associated malignancies. Blunting the acute viral load, either in terms of kinetics or absolute magnitude, may be an important strategy for preventing the excessive immune activation that causes IM. Phase II clinical testing in humans has demonstrated proof-of-principle that a gp350 subunit vaccine can prevent IM, but the mechanism by which the vaccine prevents disease remains unclear [45]. Gp350 is the major membrane glycoprotein on the virus and a major target for serum neutralizing antibodies [46]. Serum neutralizing antibodies may protect from IM by providing sterilizing immunity or by altering the speed or magnitude of viral amplification during acute EBV infection. Our studies provide evidence that targeting lytic replication can blunt viral amplification during acute infection and that lytic EBV replication may be especially susceptible to CSF-1-mediated immune responses.

For an effective EBV cancer vaccine, lower virus setpoints may translate into a decreased number of EBV-infected cells at risk for malignant transformation and a stochastic reduction in the development of EBV-associated malignancies over time. If the virus setpoint during persistent infection is linked to viral load during acute infection, then an EBV vaccine targeting lytic replication and reducing viral amplification in acute infection may be effective against both IM and EBV-associated cancers. Our studies indicate that tipping the balance in favor of host immunity against acute viral replication can alter the natural history of both acute and persistent phases of LCV infection, providing a potential vaccine strategy for protection against a spectrum of EBV-associated diseases.

## Materials and Methods

### Ethics Statement

Animal experiments were reviewed and approved by the Institutional Animal Care and Use Committee for Harvard Medical School. Animals were cared for in compliance with National Institutes of Health, US Department of Agriculture, and Harvard Medical School guidelines for animal research. Animal well-being was monitored multiple times throughout the day by animal care staff, veterinary technicians, and veterinarians, and appropriate veterinary care was provided as needed. Sedation and analgesia were administered as indicated to minimize stress and pain associated with any veterinary procedures.

### Viruses

rhLCV-infected cell lines were grown in RPMI 1640 supplemented with 10% fetal bovine serum at 37°C and 5% CO_2_. Virus stocks were derived from cell-free supernatants, and transformation titers were assayed [20]. The rhLCV strain LCL8664 [47] from which the rhLCV BAC was derived, is referred to as WT rhLCV and was obtained from the American Type Culture Collection. ΔrhBARF1 rhLCV refers to the recombinant, BAC-derived virus that has a mutated rhBARF1 open reading frame with the carboxy terminal 70 AA truncated by insertion of a premature stop codon after amino acid 150 (clone 16 rhLCV [20]). WTr rhLCV is the recombinant, BAC-derived virus with a repaired rhBARF1 open reading frame [20].

### Animal Infections

rhLCV-naïve rhesus macaques were obtained from an extended specific pathogen-free (spf) colony at the New England Primate Research Center (NEPRC). This self-sustaining breeding colony undergoes regular serologic screening to ensure that animals are free of infection from several simian viruses including herpes B, rhesus cytomegalovirus, rhesus rhadinovirus, and rhLCV. The NEPRC extended spf colony has been free of rhLCV infection for over a decade. Animals were inoculated with 10^6^ transforming units (TU) of cell-free virus applied non-traumatically throughout the oral cavity. Infection with Simian/Human Immunodeficiency Virus (SHIV) was as described [10].

### Serologic Assays

Serum antibodies against the rhLCV small viral capsid antigen (rhBFRF3) were detected by peptide immunoassays [22]. Serum antibodies against rhBZLF1, rhBRLF1, rhBMRF1, rhBALF2, rhBALF4, or rhBILF2 protein were detected by enzyme immunoassays using recombinant viral antigens [8].

### Real-time RT-PCR for rhLCV Encoded RNAs (rhEBER) and Southern Blot Analysis

Total RNA was extracted using RNA-Bee reagent (Tel-Test Inc.) on peripheral blood mononuclear cells (PBMC) or B cells affinity purified with CD20 antibody (clone 2H7, Biolegend) and CELLection Pan Mouse IgG kit (Invitrogen). Extracted RNA was reverse-transcribed using Super Script II reverse transcriptase (Invitrogen) and rhEBER173R (aaaacaggcggaccaccag) and GAPDH-R1 (gttcacacccatgacgaacatgg) primers. Real-time PCR was performed using SYBR green (Applied Biosystem). 18 ul or 0.02 ul of cDNA were amplified for 40 cycles (15 seconds at 95°C, 30 seconds at 60°C, and 30 seconds at 72°C) for rhEBER (rhEBER32F; ggaggagatgagtgtgacttaaatca and rhEBER148R; tgaaccgaagagagcagaaacc) or GAPDH (GAPDH-F; gcgagatccctccaaaatca and GAPDH-R2; ccagtggactccacgacgta), respectively. Plasmids containing rhEBER (6652–7137 nt) or GAPDH (113–510 nt, gi83641890) DNA were quantified by spectrophotometry and diluted from 10^6^ to 10^1^ copies for use as standards. PCR products were detected by gel electrophoresis, southern blot transfer, and hybridization with a ^32^P end-labeled rhEBER116R (ccaaacttttagcagcaccag) or GAPDH internal (gtggggcgatgctggcgct) oligonucleotide probe.

### Limiting Dilution Assay for Measuring Frequency of rhLCV-infected Cells in PBMC

PBMC were isolated by Ficoll-Hypaque density centrifugation and cell numbers were determined using Count Bright absolute counting beads (Invitrogen) in flow cytometry. PBMC were 2-fold serially diluted, typically at starting concentrations of 1 or 2×10^6^, and 3–5 replicates were prepared at each cell concentration. rhEBER RT-PCR was performed using the Super Script III one-step RT-PCR system (Invitrogen), rhEBER32 and rhEBER148R primers, and amplification for 35 cycles (15 seconds at 95°C, 30 seconds at 60°C, and 30 seconds at 68°C). PCR products were detected by gel electrophoresis, southern blot transfer, and hybridization with a ^32^P end-labeled rhEBER116R oligonucleotide probe. The frequency of rhLCV-infected cells in PBMC was calculated from the results of limiting dilution replicate testing using the method described by Hu, et. al. (Extreme Limiting Dilution Analysis; http://bioinf.wehi.edu.au/software/elda/) [23]. In all analyses, the single-hit hypothesis was not rejected.
